# Haplotype-resolved germline and somatic alterations in renal medullary carcinomas

**DOI:** 10.1186/s13073-021-00929-4

**Published:** 2021-07-14

**Authors:** Kar-Tong Tan, Hyunji Kim, Jian Carrot-Zhang, Yuxiang Zhang, Won Jun Kim, Guillaume Kugener, Jeremiah A. Wala, Thomas P. Howard, Yueh-Yun Chi, Rameen Beroukhim, Heng Li, Gavin Ha, Seth L. Alper, Elizabeth J. Perlman, Elizabeth A. Mullen, William C. Hahn, Matthew Meyerson, Andrew L. Hong

**Affiliations:** 1 Department of Medical Oncology, Dana-Farber Cancer Institute, Boston, MA USA; 2 Broad Institute of MIT and Harvard, Cambridge, MA USA; 3 Department of Genetics, Harvard Medical School, Boston, MA USA; 4 Department of Medicine, University of California San Francisco, San Francisco, CA USA; 5 Department of Pediatrics, University of Southern California, Los Angeles, CA USA; 6 Biostatistics and Computational Biology, Dana-Farber Cancer Institute, Boston, MA USA; 7 Public Health Sciences Division, Fred Hutchinson Cancer Research Center, Seattle, WA USA; 8 Department of Medicine, Beth Israel Deaconess Medical Center, Boston, MA USA; 9 Department of Pathology, Northwestern University, Chicago, IL USA; 10 Department of Hematology and Oncology, Boston Children’s Hospital, Boston, MA USA; 11 Department of Pediatric Oncology, Dana-Farber Cancer Institute, Boston, MA USA; 12 Department of Pediatrics, Emory University, Atlanta, GA USA; 13 Aflac Center for Cancer and Blood Disorders, Children’s Healthcare of Atlanta, Atlanta, GA USA

**Keywords:** Linked-read sequencing, Haplotypes, Renal medullary carcinoma, Sickle cell trait

## Abstract

**Background:**

Renal medullary carcinomas (RMCs) are rare kidney cancers that occur in adolescents and young adults of African ancestry. Although RMC is associated with the sickle cell trait and somatic loss of the tumor suppressor, *SMARCB1*, the ancestral origins of RMC remain unknown. Further, characterization of structural variants (SVs) involving *SMARCB1* in RMC remains limited.

**Methods:**

We used linked-read genome sequencing to reconstruct germline and somatic haplotypes in 15 unrelated patients with RMC registered on the Children’s Oncology Group (COG) AREN03B2 study between 2006 and 2017 or from our prior study. We performed fine-mapping of the *HBB* locus and assessed the germline for cancer predisposition genes. Subsequently, we assessed the tumor samples for mutations outside of *SMARCB1* and integrated RNA sequencing to interrogate the structural variants at the *SMARCB1* locus.

**Results:**

We find that the haplotype of the sickle cell mutation in patients with RMC originated from three geographical regions in Africa. In addition, fine-mapping of the *HBB* locus identified the sickle cell mutation as the sole candidate variant. We further identify that the *SMARCB1* structural variants are characterized by blunt or 1-bp homology events.

**Conclusions:**

Our findings suggest that RMC does not arise from a single founder population and that the HbS allele is a strong candidate germline allele which confers risk for RMC. Furthermore, we find that the SVs that disrupt *SMARCB1* function are likely repaired by non-homologous end-joining. These findings highlight how haplotype-based analyses using linked-read genome sequencing can be applied to identify potential risk variants in small and rare disease cohorts and provide nucleotide resolution to structural variants.

**Supplementary Information:**

The online version contains supplementary material available at 10.1186/s13073-021-00929-4.

## Background

Renal medullary carcinomas (RMCs) are rare non-clear cell renal cell carcinomas with poor clinical outcomes that disproportionately affect young individuals of African ancestry [1]. Previous studies have established an association between RMC and sickle cell trait, but the ancestral origins remain unknown [2–6]. More recently, immunohistochemistry, fluorescence in situ hybridization, sequencing following hybrid capture with targeted bait panels, and whole-exome sequencing have identified disruption of *SMARCB1*, a key component of the SWI/SNF complex, in RMC [4, 5, 7, 8]. These studies raise several questions. First, it is unknown if RMC arises from a founder population. Second, although RMC is strongly associated with sickle cell trait, it is unclear if the sickle cell mutation (Glu6Val) is the germline variant which confers risk for RMC or if there are other cooperating germline alterations in RMC. Further, even though disruption of *SMARCB1* is observed in patients with RMC, the mutational processes that drive *SMARCB1* loss in RMC remain poorly defined.

To address these questions, whole-genome sequencing is typically applied. However, whole-genome sequencing is unable to provide haplotype resolution information, and this precludes the ability to determine if large stretches of variants are occurring in *cis* or in *trans*. In contrast, both long-read and linked-read sequencing methods offer the opportunity to resolve and phase haplotype sequences [9, 10]. Linked-read genome sequencing uses barcoded high-molecular weight DNA to provide long-range information from short-read sequencing to phase germline and somatic alterations [11, 12]. This technology has been used to elucidate structural variants (SVs) and the study of long-range haplotypes [13, 14]. Here, we leveraged linked-read genome sequencing of a cohort of adolescents and young adults diagnosed with RMC who enrolled on the Children’s Oncology Group (COG) Renal Tumor Biology and Risk Classification protocol to elucidate germline and somatic alterations in RMC at the haplotype level.

## Methods

### Patient cohort

A total of 5863 patients with concern for childhood kidney cancers were enrolled on Children’s Oncology Group AREN03B2 between 2006 and 2017. Centrally reviewed pathology identified 26 patients with a diagnosis of renal medullary carcinoma. In addition, we had previously generated cell lines from 2 patients not in the COG cohort (CLF_PEDS0005 and CLF_PEDS9001) [6]. In total, samples from 15 patients’ tumor/cancer cell line with 12 matched germline DNA and 15 tumor/cancer cell line RNA samples were obtained from the COG Biopathology repository (Columbus, OH) or from prior studies through IRB approved protocols at Dana-Farber Cancer Institute and COG approved biology protocols, AREN17B2-Q, and AREN21D1-Q. Linked-read sequencing (10x Genomics) was performed on 15 tumor samples and 12 germline samples. RNA sequencing was performed on 15 tumor samples. One tumor sample (PAWMTU) failed quality control metrics for linked-read sequencing and RNA sequencing.

### Generation of sequencing dataset

Samples from COG underwent AllPrep DNA and RNA (Qiagen, Hilden, Germany) extraction based on TARGET extraction protocols [15]. For cell lines and whole-blood germline (CLF_PEDS0005 and CLF_PEDS9001) DNA extraction, we used the HMW DNA extraction kit (Qiagen) and used the protocols as outlined by 10x Genomics (Document #CG00043 Rev B). RNA extraction for these samples was performed using RNeasy (Qiagen).

Approximately 1 μg of genomic DNA and 1 μg of tumor DNA were subjected to size selection when concentrations were above > 0.4 ng/μL. In cases where samples did not meet this threshold, no size selection was performed. Samples were subjected to linked-read genome library preparation (10x Genomics, Pleasanton, CA) and sequenced with a NovaSeq 6000 (Illumina, La Jolla, CA). We achieved 43.3× mean coverage of normal samples and 82.6× mean coverage of tumor/cancer cell line samples.

Approximately 0.5 μg of RNA was subjected to RNA sequencing using TruSeq v2 chemistry and sequenced on a NovaSeq (Illumina). Samples with > 50 million reads were used for subsequent analysis (13 of 15 patients) after alignment with STAR [16].

### Conversion of BCL files to Fastq files

BCL files generated from the sequencing run were demultiplexed and converted into fastq files using the Long Ranger mkfastq tool (Version 2.1.6) provided by 10x Genomics (https://support.10xgenomics.com/genome-exome/software/downloads/latest) [17]. This is a wrapper program around the Bcl2fastq2 (version 2.17.1.14) program provided by Illumina. The longranger mkfastq program was run with the parameters --ignore-dual-index and --delete-undetermined to generate the .fastq files from the BCL files.

### Long Ranger mapping and germline variant calling

The full Long Ranger pipeline was executed on the full set of fastq files corresponding to each sample, using the Long Ranger WGS program (version 2.2.2) provided by 10x Genomics with the command “longranger wgs.” The Long Ranger-compatible GRCh38 reference genome (GRCh38 Reference - 2.1.0; Sep 15, 2016) was obtained from http://cf.10xgenomics.com/supp/genome/refdata-GRCh38-2.1.0.tar.gz [18] and used as a reference genome for alignment and germline variant calling. As multiple sequencing runs were used to sequence a single sample, all runs corresponding to each sample were specified within the .mro configuration file. The following parameters were also specified within the .mro configuration file used in the Long Ranger run: “bc_in_read”: 1, “bc_length”: 16 “sample_indices”: [“any”].

### Generation of phased germline calls

Germline variant calling was performed with the genomic analysis toolkit (GATK version 4.0.10.0) [19] within the Long Ranger pipeline by specifying it as the variant caller using the “vc_mode” argument in the .mro configuration file. This enabled germline variants to be phased into haplotype blocks based on sequence barcodes obtained by linked-read genome sequencing. Detected variants with a phase quality of ≥ 23 were deemed reliable for assignment into a single haplotype block.

### Somatic single-nucleotide variation (SNV) calling

Somatic SNVs calling was performed as previously described [20, 21]. MuTect (version 1.1.4) was used to identify somatic SNVs in the 12 samples for which matched tumor or cell line and germline DNA were available [22]. Candidate sites with the “KEEP” tag were then retained. In order to account for systematic artifacts caused by mapping, sequencing errors, and common germline SNPs, we used MuTect to generate a Panel of Normals (PON) from the Long Ranger-aligned normal samples in our study. Variant calling was performed on all 12 normal samples in our cohort in the “single sample mode,” using the parameter “–artifact_detection_mode.”

We then applied a set of filters. First, we removed systematic artifacts found in ≥ 2 samples in the PON. We retained variants of allele frequency > 10% and with ≥ 6 variant reads, as previously described [20, 21]. We subsequently removed variants present in the 1000 Genomes or gnomAD databases at allele frequency > 0.1% [23, 24]. For the 3 samples lacking a matched germline sample, we used MuTect in the “–artifact_detection_mode” to call possible somatic SNVs. Subsequent filters were as for the matched samples.

### Copy number analysis

TITAN (version 1.15.0) was used to identify copy number alterations [25]. Each tumor-normal sample pair was fed into the TITAN software where the coverage profile information for each 10-kb bin was evaluated for each bam file. SNP calling was performed on a list of sites found in the dbSNP database [26] on the normal sample to identify heterozygous sites. These heterozygous sites were then used to calculate the B-allele frequency for the corresponding tumor sample. Following GC correction, coverage profile information and B-allele frequency information were then fed into the TITAN statistical model for inference of copy number state. Ploidy = {2,3} and tumor clusters = {1,2} were used as default parameters, to generate four possible solutions based on the data. Each solution was manually curated to establish the most reasonable parameters for each dataset. All samples were found to be of ploidy = 2, and samples with either 1 or 2 tumor clusters were detectable. For the 3 samples lacking a matched germline sample, the germline sample of CLF_PEDS0005 served as the match normal. Each solution was manually curated, and the copy number profile assessed for the presence of deletions on chromosome 22 where *SMARCB1* resides.

### Structural variant calling

We used SvABA (version 1.1.2) to identify somatic structural variants [27]. Somatic SV calling was performed using the bam files corresponding to each pair of tumor and normal samples. Candidate somatic structural variants with the “PASS” tag were retained for further analysis. For the 3 samples lacking a matched germline sample, SvABA SV calling was performed on the tumor sample alone. A small fraction of SV calls related to *SMARCB1* loss were rescued from the unfiltered list, as they did not meet the default detection threshold of SvABA. Notably, all structural variants associated with *SMARCB1* loss events were manually curated in the Integrative Genomics Viewer (IGV) to ensure call reliability. Soft-clipped sequences found at each of the breakpoints for the SV events were remapped using BLAT in IGV to ensure accurate mapping to the other breakpoint.

### Annotation of variants identified

Annovar (version 2018-04-16) was used to annotate all SNVs, indels and breakpoints corresponding to structural variants [28]. Each variant was annotated using the Annovar databases refGene, knownGene, ensGene, dbnsfp35c, dbscsnv11, cosmic70, esp6500siv2_all, exac03, avsnp150, clinvar_20190305, 1000g2015aug_all, 1000g2015aug_afr, genomicSuperDups, and gwasCatalog.

### Inferring sex of patients from genome sequencing

To infer the sex of each patient in our cohort, the number of reads mapped to each chromosome was counted using samtools idxstats [29]. The total read count for each sample was obtained by tallying reads in chr1-22, chrX, chrY, and chrM. These data were used to calculate for each sample the percentage of reads arising from each chromosome, and the ratio of chrX to chrY reads. We determined samples with chrX to chrY ratio of ~ 40:1 to be female and 4:1 to be male.

### 1000 Genome Project phase 3 genotype calls

Genotype information or corresponding ancestry information from the 1000 Genomes Project [24] database in the hg38 coordinates (liftover) was downloaded from the 1000 Genomes Project website (http://ftp.1000genomes.ebi.ac.uk/vol1/ftp/release/20130502/supporting/GRCh38_positions/ and ftp.1000genomes.ebi.ac.uk/vol1/ftp/technical/working/20130606_sample_info/20130606_sample_info.xlsx) [24]. Among the 1000 Genomes Project cohort, 137 individuals were found to be heterozygous for the sickle cell mutation (rs334). These samples were subsequently used for the haplotype level analysis.

### Analysis of ancestry for RMC patients

To establish the ancestry of each RMC patient, we compared our samples to 2504 samples of defined ancestry from the 1000 Genomes Project (phase 3) [24]. Germline SNP calls for both 1000 Genomes Project samples and RMC samples were converted from VCF format into Plink .bed format and then merged. Principal component analysis of these samples was performed with Plink (v1.90b6.18) [30] using a list of 6,513,809 variants detected in the RMC cohort, with a minor allele frequency of ≥ 0.05 and missing call frequency < 0.1 in the combined cohort, similar to previous studies (https://github.com/GerkeLab/TCGAancestry) [31]. We manually inspected the first six principal components and found that principal components 2 and 3 (PC2 and PC3, respectively) can effectively separate the 1000 Genomes Project samples into the five major populations. The ancestry of each RMC sample was then inferred based on how it clustered with each of these five major populations in the PCA plot.

### Haplotype principal component analysis of sickle cell allele

To perform haplotype principal component analysis of the region corresponding to the sickle cell allele, we used the Long Ranger pipeline (version 2.2.2) provided by 10x Genomics to phase germline calls and to generate haplotypes. Specifically, we analyzed all variant alleles flanking ±100 kb of the sickle cell mutation in all 14 patient samples of African ancestry. For cases lacking the germline sample (n = 3), we used germline calls from the tumor samples. Only variants represented in the 1000 Genomes Project database were analyzed to ensure comparability of the variants in both the RMC and the 1000 Genome cohorts and to remove sites with poor genotype calls from the RMC samples. We also inspected the “PS” tags of all variants within this 200-kb region to ensure the calling of variants in this region as a single continuous haplotype. In two of these cases (PEDS0005T and PAWMTU), single breaks were observed in the haplotypes generated from the normal samples, though these could be stitched together using haplotypes generated from the matched tumor samples.

Having established the germline variants for analysis, and the contiguity of the haplotypes in this 200-kb region for the RMC patient samples, the phased germline calls of each RMC patient were defined as one of two haplotypes, the sickle cell mutant haplotype or the wild type haplotype, based on the presence or absence of the HbS allele. Haplotypes obtained from the 1000 Genomes Project (phase 3) [24] individuals with the sickle cell mutation were similarly defined as mutant or wild-type haplotype. These haplotypes of RMC patients and 1000 Genome patients were compiled, represented as vectors of “zeroes” (reference allele) and “ones” (variant allele), and merged into a single matrix.

We then performed principal component analysis using R (version 3.5.0) on the haplotype vector (of “zeros” and “ones”) corresponding to each sample and for both the sickle cell mutant and non-mutant haplotypes. The position of each haplotype was displayed on the first two principal axes as indicated.

### Fine-mapping of sickle cell haplotype

As the Long Ranger germline variant caller analyzes one sample at a time and does not output phased genotype calls for homozygous reference sites by default, sites which are variant at one coordinate in a particular sample may not possess a genotype call in a different sample. Thus, the genotype states for a number of sites can be missing when genotype calls from multiple samples are merged together. Typically, most of these sites with an absent genotype call are homozygous reference sites, and can be imputed accordingly. Nonetheless, to ensure the accuracy of genotype calls for our fine-mapping analysis, joint variant calling was simultaneously performed on all samples using GATK (version 4.0.10.0) [32]. Joint variant calling identified 1979 variants, of which 1715 (86.7%) were concordant with variants identified by per-sample variant calling. Most non-concordant variants were small indels that were difficult to assign to a single genomic coordinate. The 1715 concordant variants were then subjected to fine-mapping analysis.

Fine-mapping was performed to identify possible additional candidate risk variants in linkage disequilibrium with the sickle cell mutant allele. Specifically, the PS tag was used to identify all variants in the same haplotype block within the 200-kb region surrounding the sickle cell mutation. The strong association between sickle cell trait and RMC argued that the variant conferring risk for RMC most likely resides within the haplotype encompassing the sickle cell mutation, but not the corresponding wild-type haplotype at the same locus. Based on this principle, we then searched for all variants significantly over-represented in the sickle cell mutant haplotype as compared to the wild-type haplotypes in the same patients. This was done by enumerating the number of times each variant was observed in the haplotype blocks containing or lacking the sickle cell mutation. Significant over-representation of any variant in the sickle cell haplotype vs. the wild-type haplotype was tested by one-sided Fisher’s exact test using R (version 3.5.0).

### Identification of potentially deleterious germline variants

**Fig. 1:**
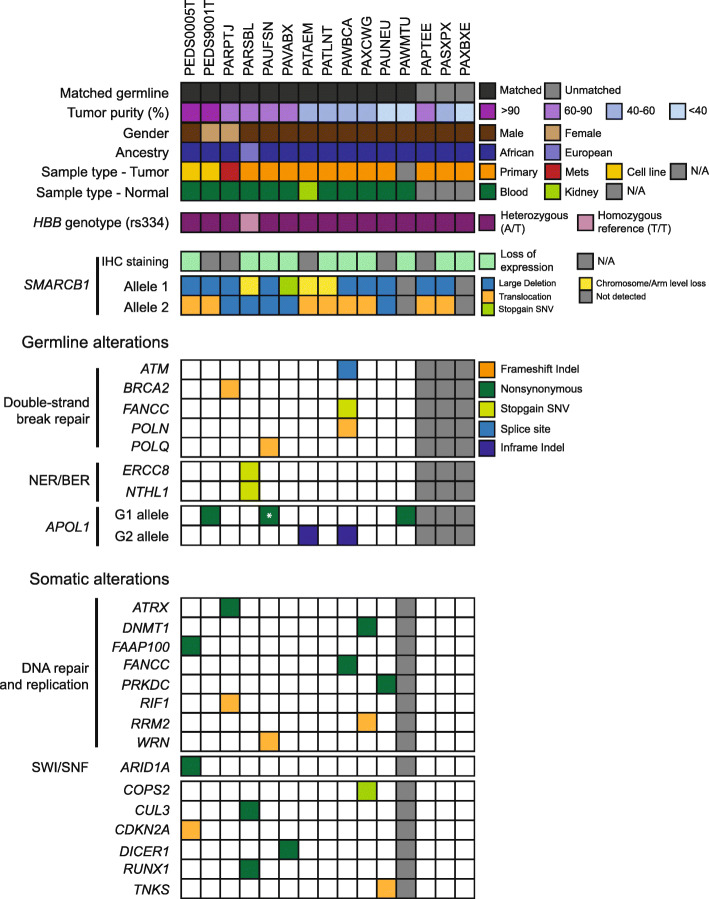
Germline and somatic alterations detected in renal medullary carcinoma. Co-mutation plot depicting the germline and somatic alterations identified in each RMC sample in the Children’s Oncology Group cohort as indicated. The detailed information corresponding to each sample is indicated in the top panel, followed by the *HBB* genotype and the type of somatic *SMARCB1* alteration in each allele. Expression of SMARCB1 as assessed by immunohistochemistry (IHC) staining is also as indicated. Germline alterations (i.e., frameshift indels, stop-gain, splicing mutations) in the double-strand break repair, nucleotide excision repair (NER), and base excision repair (BER) pathways are shown, followed by the status of the G1/G2 risk alleles of the *APOL1* kidney disease predisposition gene. Somatic alterations in the DNA repair and replication pathway, SWI/SNF complex, and other cancer-related genes are displayed in the bottom panel. *Sample which is homozygous for the *APOL1* G1 allele

We first identified 952 candidate genes based on prior studies of germline variants or genes affecting DNA repair [33–35]. We used the germline Long Ranger output based on GATK [19]. We filtered out poor-quality reads and annotated these calls with reference to the 1000 Genomes Project or gnomAD [23, 24]. We then focused on disruptive events (e.g., splice variants, frameshift indels or deletions, and nonsense mutations) with population allelic frequency < 0.1% and manually curated candidate germline variants presented in Fig. 1 using the Integrative Genomics Viewer [36].

### Identification of potentially relevant somatic variants

We searched the filtered list from the above SNV calling methods for COSMIC-nominated mutations [37], nonsynonymous or stop-gain mutations that had a somatic variant allele frequency (VAF) > 10% while having a population allelic frequency (AF) < 0.1% in the 1000 Genomes Project African population. We then used mSigDB to identify variants significant in cancer biology [24, 38].

### Analysis of microhomology at DNA breaks

To assess the patterns of microhomology at each of the translocation and large deletion events associated with *SMARCB1* loss events, the fusion sequence for each SV event was reconstructed based on the SV calls from SvABA [27]. These sequences were then compared with the reference sequences at both breakpoints to detect any inserted sequences. Reference sequences at both breakpoints were also searched for sequence microhomology close to the breakpoints.

### Annotation of repeat elements at DNA breaks

The hg38 RepeatMasker track was downloaded from the UCSC Genome Browser [39] using the Table Browser functionality on the website (Group: Repeats, track: RepeatMasker) in bed format. Using the “intersect” function in bedtools (v2.28.0) [36], we identified the potential overlap between DNA breakpoint sites identified in our study and repeat elements in the RepeatMasker database [40].

### RNA sequencing expression analysis

RNA was extracted with Allprep DNA and RNA as above. Samples were sequenced with an average of 135 million reads per sample. Following sequencing, samples were mapped, aligned, and quantified using the same algorithms for the Cancer Cell Line Encyclopedia and Genotype-Tissue Expression [41, 42]. From RSEM quantified samples, DESeq2 was used to perform normalization and differential expression analyses of the *SMARCB1* gene [43, 44].

### Analysis of fusion transcripts at *SMARCB1*-related breakpoints

Fusion transcripts associated with *SMARCB1*-related breakpoints were identified by manual assessment of each *SMARCB1*-associated breakpoint in the Integrative Genomics Viewer [36]. For each pair of breakpoints corresponding to a large deletion or translocation event, the region retained subsequent to the structural variant event was carefully examined for reads that were discordantly mapped or were soft-clipped in the RNA sequencing dataset. Particular attention was paid to genes which overlapped with the breakpoints of interest. Any identified soft-clipped sequences were remapped by BLAT to identify the partner region to which fusion transcripts were linked [45]. All identified soft-clipped sequences were mapped to the retained genomic region on the other side of the breakpoint. Each such fusion junction was carefully examined for the presence of the 5′-splice site (GT) and 3′-splice site sequence (AG) to establish transcript orientation.

## Results

### Landscape of germline and somatic alterations in RMC

A total of 5863 children and adolescents with renal masses in North America were enrolled in the COG AREN03B2 renal tumor biology study between 2006 and 2017. This represents an estimated 90% of all kidney cancers seen in children and adolescents. The twenty-six patients enrolled had a central pathology diagnosis of RMC. From these 26 patients, 14 tumor samples and 12 germline samples representing 15 unique patients (13 male and 2 female) met the quality control metrics following linked-read genome sequencing (10x Genomics) and RNA sequencing (Fig. 1, Additional file 1: Tables S1-S2, the “Methods” section) [13]. Of these 15 patients, 10 had SMARCB1 immunohistochemistry performed and described loss of SMARCB1 (Fig. 1, Additional file 2: Fig. S1a, Additional file 1: Table S1). Notably, the male bias we observed (87%) in our study is similar to three prior studies (70–77%) on RMC [46–48]. We achieved a mean sequencing coverage of 43.3× for the germline samples and 82.6× for the tumor samples. Further, germline and somatic variants were phased into haplotype blocks with a median N50 phase block size of 2.2 Mbp for the normal DNA and 1.3 Mbp for the tumor DNA in this study (Additional file 1: Table S3).

To establish whether there are any cooperating germline alterations in RMC, we analyzed the germline variants (Fig. 1; Additional file 2: Fig. S1b). We first compared our samples with populations from the 1000 Genome Project using principal component analysis (PCA) to determine the ancestry. We found that 14 patients were of African origin and 1 patient was of European origin (Fig. 1, Additional file 2: Fig. S1c) [24]. Subsequently, we assessed genes implicated in cancer predisposition syndromes [33, 34]. Notably, we had chosen this candidate gene approach in the analysis of germline variants due to the small cohort size of our study (n = 15) which has inadequate statistical power to perform a powered unbiased genome-wide search for new candidate genes. We identified 7 heterozygous germline alterations in the double-strand break, nucleotide excision, and base excision repair pathways (Fig. 1, Additional file 1: Table S4). We then assessed alleles affecting *APOL1* function which are associated with chronic kidney disease (CKD) in African Americans [49]. Four patients were heterozygous for G1 alleles (comprising two variants) or the G2 allele in the *APOL1* gene while 1 patient (PAUFSN) was homozygous for the G1 alleles (the homozygous state primarily confers increased risk for CKD). Allelic frequency in African Americans for the G1 alleles at chr22:36265860 is 0.2189 and at chr22:36265988 is 0.2204 while the G2 allele at chr22:36265995 is 0.1357 [23]. Taken together, we did not identify any recurrently mutated genes affecting the DNA repair pathway or chronic kidney disease in the germline of RMC patients.

We then assessed the landscape of somatic alterations in RMC by integrating copy number analysis, structural variant calling, indel analysis, and SNV calling in 11 tumors with matched germline samples and in 3 tumors without matched germline samples (Fig. 1, the “Methods” section). We found an average of 39.2 exonic somatic SNVs and a mutation rate of 1.14 per Mbp in RMC samples (Additional file 2: Fig. S2a). We identified copy number loss on chromosome 22q in 11/11 samples with matched germline samples and 2/3 without matched germline samples (Additional file 2: Fig. S2b-c). The next most common copy number alteration was copy number gain on chromosome 8q in 3/11 samples with matched germline samples and 0/3 samples without matched germline samples. Powered by linked-read genome sequencing, we found that 100% of evaluable tumors had bi-allelic somatic disruption of the *SMARCB1* gene on both parental haplotypes (Fig. 1, Additional file 1: Table S5). We identified somatic variants (VAF ranging from 0.11 to 0.42) involving DNA repair and replication, the SWI/SNF complex (*ARID1A*), and other cancer-related genes, but none were recurrently altered (Fig. 1, Additional file 1: Table S6). Together, our data suggests that the genomes of RMC have few alterations and that there are no other high-frequency recurrently mutated genes beyond *SMARCB1* in RMC.

### The HbS allele in RMC patients is derived from multiple ancestries

**Fig. 2:**
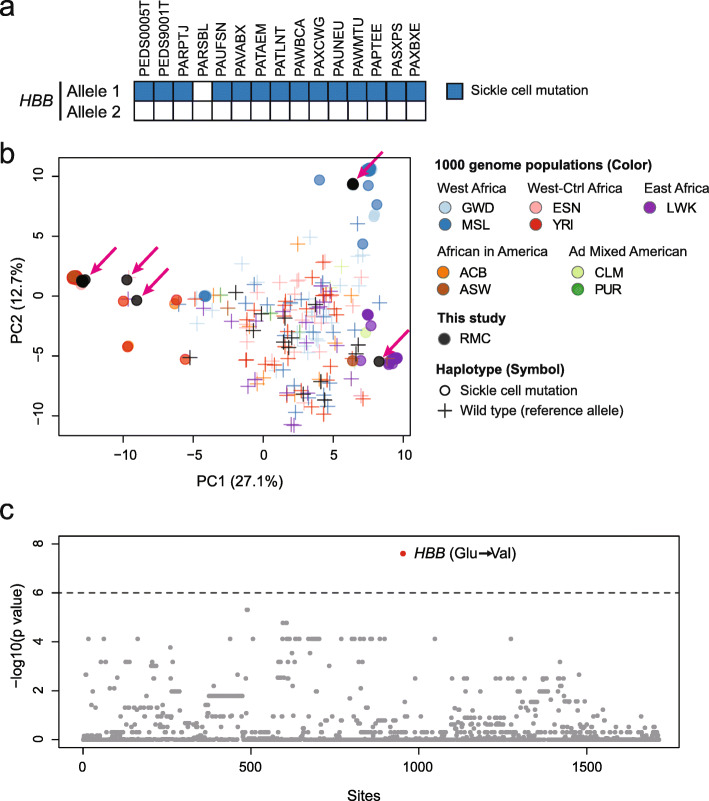
RMCs originate in patients with diverse sickle cell haplotypes. **a** Heatmap depicting the mutation status of each *HBB* allele in RMC patients. Patients carrying the canonical Glu6Val (rs334) sickle cell mutation are as indicated. **b** Principal component analysis of haplotypes carrying the sickle cell mutation or the wild-type *HBB* allele in RMC patients. Sickle cell trait and reference haplotypes corresponding to different populations from the 1000 Genome database are also depicted for comparison. The percentage of variation explained by each principal component (PC) is as indicated. Arrows in magenta highlight the sickle cell mutant haplotypes of RMC patients which are broadly separated into three different population groups. Population codes represented in the figure are as follows: ACB, African Caribbeans in Barbados; ASW, Americans of African ancestry in southwest USA; CLM, Colombians from Medellin, Colombia; ESN, Esan in Nigeria; GWD, Gambian in Western Divisions in the Gambia; LWK, Luhya in Webuye, Kenya; MSL, Mende in Sierra Leone; PUR, Puerto Ricans from Puerto Rico; and YRI, Yoruba in Ibadan, Nigeria. Note that numerous sickle cell mutant haplotypes were nearly identical, causing them to appear as overlapping points on the plot. **c** Fine-mapping analysis was performed to identify the variants that are significantly enriched in the sickle cell mutant haplotypes but depleted in the reference haplotypes for variants sorted by genomic coordinates. The dotted line represents a p-value of 10^−6^

We then assessed the sickle cell mutation allele (HbS; Glu6Val) to determine the sickle cell mutation status of patients with RMC. Fourteen of 14 individuals of African ancestry had sickle cell trait whereas the individual of European ancestry did not harbor the HbS mutation (Fig. 1, Fig. 2a, Additional file 2: Fig. S1c and Additional file 1: Supp. Table S7). We then searched the HbVar database for alterations in other hemoglobin genes [50]. We did not identify co-occurrence of alpha thalassemias, but we found several variants of unknown significance in *HBE*, *HBM*, and *HBQ1* in 2 patients (Additional file 1: Table S8). Thus, our results suggest that the majority of RMC patients are carriers of the sickle cell mutation without a cooperating hemoglobinopathy.

To evaluate for a possible founder population in RMC given the association with the sickle cell trait, we asked if we could observe a shared haplotype among these unrelated RMC patients at the sickle cell locus. We reconstructed the haplotype in the 200-kb region surrounding *HBB* using linked-read sequencing. For comparison, we included phased haplotype calls of individuals with sickle cell traits from the 1000 Genomes Phase 3 Project [24]. We performed haplotype-level PCA for these two groups. We found that the haplotypes containing the HbS mutation were divided into three distinct groups unrelated to a diagnosis of RMC (Fig. 2b). These groups were tightly clustered within themselves suggesting that the haplotypes in each cluster were shared within each group. Moreover, these groups represent three major geographic regions in Africa, namely West Africa (top-right cluster—GWD and MSL), West-Central Africa (left cluster—ESN and YRI), and East Africa (bottom-right cluster—LWK). In contrast, these differences were not observed in the corresponding wild-type *HBB* haplotype (Fig. 2b). We performed PCA using unphased genotype data for different region sizes around the sickle cell mutation and found that these sub-population-specific differences extended to 2 Mbp (Additional file 2: Fig. S3a-c). These results suggest that the risk allele underlying RMC arises from diverse ancestries rather than a founder population.

To assess whether the association between RMC and sickle cell trait is due to the HbS allele or to another variant in strong linkage disequilibrium with it, we performed fine-mapping of the sickle cell locus (Fig. 2c). We hypothesized that if another candidate variant exists, then it should be significantly over-represented in the HbS haplotype compared to the wild-type haplotype. We see that the sickle cell mutation was the most significantly (p-value = 2.5 × 10^−8^) over-represented in the HbS haplotypes among the 14 patients of African ancestry. The next three most highly over-represented variants (p-value = 4.9 × 10^−6^) were common variants (> 18% in the African population), and all three were observed in *cis* with HbS in gnomAD or the 1000 Genomes Project [23, 24]. Notably, these intergenic variants did not co-localize with H3K27Ac peaks or DNase I peaks, suggesting that they are unlikely to reside in enhancer elements affecting *HBB* or other genes [51]. Taken together, our results provide evidence to suggest that the sickle cell mutation is a germline variant that confers risk for RMC.

### Patterns of structural variants driving *SMARCB1* loss in RMC

**Fig. 3:**
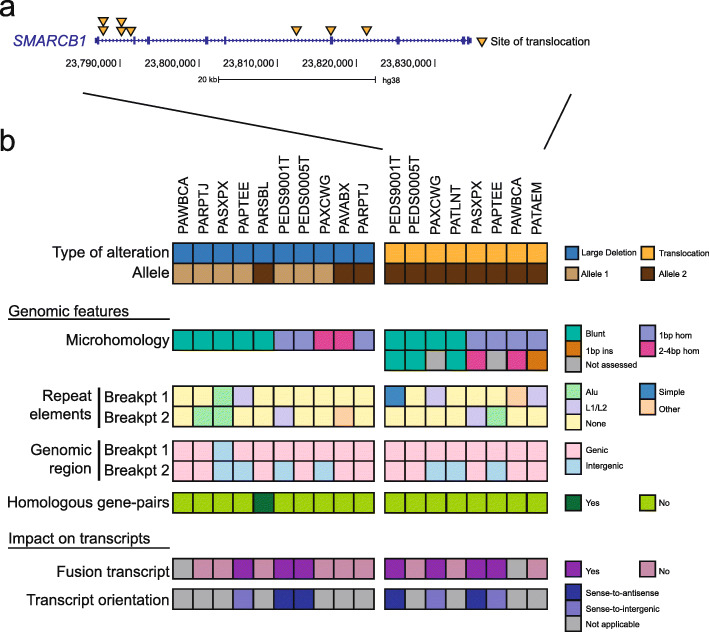
Features of breakpoints associated with *SMARCB1* structural variants. **a** Schematic depicting the sites of DNA breaks corresponding to *SMARCB1* translocations. The two DNA break hotspots in the *SMARCB1* gene are as indicated. **b** Heatmap depicting the *SMARCB1* alteration identified in each allele, the genomic features associated with each *SMARCB1*-related breakpoint, and the impact of these alterations on the creation of fusion transcripts. Top: the type of *SMARCB1* alteration identified in each allele. Middle: genomic features associated with each *SMARCB1*-associated breakpoints. Whether the breakpoint localizes to a genic/intergenic region, whether or not the breakpoint is transcribed, the type of repeat element which overlaps with the breakpoint, whether the pair of breakpoints occurs on a pair of homologous genes, and the number of base-pairs of microhomology sequence detected at the site of DNA fusion are as indicated. Bottom: the impact of *SMARCB1*-associated deletions and translocations on the occurrence of fusion transcripts

We assessed the spectrum of alterations by which *SMARCB1* was disrupted. We found structural variant (SV) events in 22 of 26 alleles from 13 patients (85%) (Fig. 1). Fourteen SVs were deletions (median deletion size of 3.5 Mbp), 8 were balanced translocations, and the remainder were either chromosome/arm losses (n = 3) or a stop-gain mutation (n = 1) (Additional file 1: Table S5). Among the 8 *SMARCB1* translocations, 5 occurred within the first intron. Four of these *SMARCB1* breakpoints were found in close proximity (< 100 bp) to each other suggesting that these sites might represent hotspots of DNA breakage (Fig. 3a). However, we did not identify recurrent partner translocations (Additional file 2: Fig. S4a-b). We further identified several instances of kataegis occurring on chromosome 22 where *SMARCB1* resides (Additional file 2 Fig. S5a-c) [52]. In summary, the loss of *SMARCB1* in RMC is driven primarily through SVs.

We then assessed genomic features of SVs associated with *SMARCB1* loss. Analysis of the 24 evaluable fusion events (deletions and translocations) showed these SVs had either blunt end assembly (12/24) or 1-bp microhomologies (7/24) (Fig. 3b, Additional file 1: Tables S9-S10). This suggests that these sites were repaired by classical non-homologous end joining (c-NHEJ) [53, 54]. We next assessed for repeat elements at the breakpoints. We found these events were rare, with 5 harboring a L1/L2 repeat element and 4 with an Alu element. In addition, 26/34 breakpoints occurred in genic regions suggesting a possible relationship between transcription and acquisition of DNA breaks. We then looked at the genes involved in these breakpoints and found that 3 occurred in the calcineurin-binding protein gene, *CABIN1*. Two additional breakpoints were found in the breakpoint cluster region gene, *BCR*. We further noted that in the PARSBL sample, a pair of breakpoints occurred in *GGT1* and *GGT2* (sequence identity = 97%) suggesting a relationship between homologous gene sequences and the occurrence of a SV. Together, our findings highlight a range of genomic features that may be involved in driving *SMARCB1* loss in RMC.

We then asked if these SV events associated with *SMARCB1* loss might also be driving the formation of fusion transcripts. We found fusion transcripts in 3 of 9 deletion events and in 4 of 7 translocation events. Four of these 7 fusion transcripts were generated either in the sense-to-antisense orientation of annotated genes, and the remainder were generated in the sense-to-intergenic orientations suggesting that these would not lead to a functional fusion protein. Together, our work indicates fusion transcripts are frequently generated as by-products of *SMARCB1* loss events and are unlikely to promote tumorigenesis.

## Discussion

The sickle cell trait has been associated with renal medullary carcinoma and has been proposed as a risk locus for this disease [2]. It has remained unproven if another variant in this region could predispose a patient to RMC or if the HbS allele is the sole germline risk allele. Here, we used linked-read genome sequencing to reconstruct the haplotypes surrounding the hemoglobin locus. We found that the HbS allele in patients with RMC derives from three sub-populations within Africa suggesting that there is not a founder effect from the sickle cell mutation. Furthermore, fine-mapping of this region did not identify another allele associated with RMC. Rather, the sickle cell mutation is the strongest candidate in this region. We then expanded our search to the germline and did not find recurrent alterations in cancer predisposition genes or genes affecting kidney injury. For instance, although 3 of 12 (25%) and 2 of 12 assessable patients (17%) had the G1 and G2 *APOL1* alleles, respectively, the frequencies of these alleles which are linked to the risk of kidney damage [49] were not elevated in comparison with the general African American population (22% and 14%, respectively). Taken together, our study provides further evidence to support the concept that sickle cell mutation is the main germline risk factor underlying RMC.

Our study of samples from the 11-year COG experience of children and adolescents with RMC confirms that the tumor suppressor gene, *SMARCB1*, is primarily disrupted through structural variants such as deletions and translocations. These results add to the growing body of literature reporting an array of mechanisms by which *SMARCB1* is disrupted in RMC [4, 5, 8, 55] and in other cancers [56–58]. Using linked-read genome sequencing, we found that these structural variants likely are repaired by c-NHEJ, concordant with SVs observed in other cancer types [53]. While an issue with DNA damage repair might help drive the occurrence of *SMARCB1* associated SVs, we did not identify any recurrent germline alterations in the double-strand break and NER/BER pathways nor recurrent somatic alterations in genes related to DNA repair and replication. Our findings of a predisposing and potentially causal allele in patients with cancer of low somatic mutation burden raise questions around the environment of the precursor cells. For example, nicotinic acetylcholine receptor risk alleles are associated with increased tobacco smoking and increase the carcinogenic risk to precursor lung cells [59]. Similarly in RMC, the HbS allele may confer replication stress or chronic inflammation which may lead to an increased risk of double-stranded breaks in precursor kidney cells [60, 61].

Our results also illustrate how germline alleles conferring disease risk such as the HbS allele can be identified in rare diseases using linked-read genome sequencing in unrelated individuals. While co-segregation and genetic linkage in families and relatives of a specific cancer are typically applied to identify the genes responsible for cancer predisposition, difficulties in collecting these samples have prevented these approaches from being applied in the context of rare diseases like RMC. Nonetheless, unrelated individuals with the same disease often also share long tracts of genetic material in the region responsible for disease risk. By applying this principle, identity-by-descent (IBD) mapping has identified major loci for serum triglycerides, schizophrenia, multiple sclerosis, *BRCA1*, and a founder population with *TP53* R337H [62–68]. However, accurate reconstruction of haplotypes normally requires related individuals [69]. Absent a related cohort, statistical-based approaches have been used but may miss rare variants [30, 70–75]. Furthermore, some of these approaches require large cohorts to achieve adequate statistical power while others require a representative panel of reference haplotypes. Therefore, our study illustrates how linked-read genome sequencing can overcome these challenges and identify potential predisposition loci in rare cohorts.

Our study however has a few limitations. While the HbS allele has been identified as a strong risk allele for RMC patients in our study, it has not been demonstrated experimentally that this allele plays a causative role in RMC. One approach to consider would be to evaluate RMC development in large cohorts of HbS mutant mice in combination with other genetic alterations and/or cancer-promoting environments. While a strong association of RMC with individuals of African ancestry was observed, our study is unable to confirm if this is driven purely by genetics (e.g., the HbS allele) or due to epidemiologic, environmental, and/or other ethnicity-specific differences. It should nonetheless be noted that all patients in our cohort were obtained from North America, where environmental and epidemiologic differences are likely minimized. We also observed 10.3-fold (p = 4.87 × 10^−15^) more individuals with sickle cell trait in RMC patients of African ancestry than we might expect by chance among African Americans. Thus, while ethnicity-specific epidemiologic and environmental differences might contribute in part to the incidence of RMC, ethnicity-specific genetic differences likely play an important role in RMC. Finally, due to low tumor purity, we did not identify mutations or deletions/translocations in the *SMARCB1* gene despite the loss of SMARCB1 by immunohistochemistry in PAWMTU and PAXBXE.

## Conclusions

We have shown here that technologies such as linked-read genome sequencing can significantly enhance our ability to resolve the genetic basis underlying rare diseases. Specifically, we find that the HbS allele is likely the germline allele conferring risk for RMC and does not arise from a single founder population. We further show at nucleotide resolution that the disrupting SVs observed in disrupting *SMARCB1* is likely repaired by non-homologous end-joining. The combined analysis of germline and somatic genome alterations in cancer, together with haplotype-resolved sequencing, may also reveal links between the impact of germline mutations such as HbS that create a cancer-promoting tissue environment and mutations in genes such as *SMARCB1* that arise in this environment.

## Supplementary Information

**Additional file 1: Tables S1-S10.** Supplementary tables include the following: clinical info (Table S1), inferred sex (Table S2), Longranger statistics (Table S3), germline variants (Table S4), *SMARCB1* SVs (Table S5), somatic variants (Table S6), sickle cell locus (Table S7), *HBB* variants (Table S8), breakpoint repeats (Table S9), and breakpoint microhomology (Table S10). Legends for each table are included at the top of each table in the Microsoft Excel file.

**Additional file 2: Figures S1-S5.** Supplementary figures and their corresponding figure legends.

## Data Availability

Sequencing data reported in this paper has been deposited in the database of Genotypes and Phenotypes (dbGaP) under study accession phs001800.v2.p1 at https://www.ncbi.nlm.nih.gov/projects/gap/cgi-bin/study.cgi?study_id=phs001800.v2.p1 [76]

## Abbreviations

ACB: African Caribbeans in Barbados
AFR: Africans
AMR: Admixed Americans
ASW: Americans of African ancestry in southwest USA
BER: Base excision repair
c-NHEJ: Classical non-homologous end-joining
CKD: Chronic kidney disease
CLM: Colombians from Medellin, Colombia
COG: Children’s Oncology Group
EAS: East Asian
ESN: Esan in Nigeria
EUR: Europeans
GATK: Genome Analysis Toolkit
GWD: Gambian in Western Divisions in the Gambia
IBD: Identity-by-descent
IGV: Integrative Genomics Viewer
LWK: Luhya in Webuye, Kenya
MSL: Mende in Sierra Leone
NER: Nucleotide excision repair
NHEJ: Non-homologous end-joining
PC: Principal component
PCA: Principal component analysis
PON: Panel of Normals
PUR: Puerto Ricans from Puerto Rico
RMC: Renal medullary carcinoma
SAS: South Asian
SNV: Single-nucleotide variation
SV: Structural variants
SWI/SNF: Switch/sucrose non-fermentable
TARGET: Therapeutically Applicable Research to Generate Effective Treatments
YRI: Yoruba in Ibadan, Nigeria

## References

[1] Ezekian B, Englum B, Gilmore BF, Nag UP, Kim J, Leraas HJ, Routh JC, Rice HE, Tracy ET (2017) Renal medullary carcinoma: a national analysis of 159 patients. Pediatric blood & cancer. 10.1002/pbc.26609 [doi] 10.1002/pbc.26609 28485059

[2] Davis CJ, Mostofi FK, Sesterhenn IA (1995). Renal medullary carcinoma. The seventh sickle cell nephropathy The American Journal of Surgical Pathology.

[3] Calderaro J, Moroch J, Pierron G (2012). SMARCB1/INI1 inactivation in renal medullary carcinoma. Histopathology.

[4] Calderaro J, Masliah-Planchon J, Richer W (2016). Balanced translocations disrupting SMARCB1 are hallmark recurrent genetic alterations in renal medullary carcinomas. Eur Urol.

[5] Msaouel P, Malouf GG, Su X (2020). Comprehensive molecular characterization identifies distinct genomic and immune hallmarks of renal medullary carcinoma. Cancer Cell.

[6] Hong AL, Tseng Y-Y, Wala JA, et al (2019) Renal medullary carcinomas depend upon SMARCB1 loss and are sensitive to proteasome inhibition. Elife. 10.7554/eLife.44161 10.7554/eLife.44161 PMC6436895 30860482

[7] Cheng JX, Tretiakova M, Gong C, Mandal S, Krausz T, Taxy JB (2008). Renal medullary carcinoma: rhabdoid features and the absence of INI1 expression as markers of aggressive behavior. Modern Pathology: an Official Journal of the United States and Canadian Academy of Pathology, Inc.

[8] Carlo MI, Chaim J, Patil S, et al (2017) Genomic characterization of renal medullary carcinoma and treatment outcomes. Clinical Genitourinary Cancer. https://doi.org/S1558-7673(17)30096-4 [pii] 10.1016/j.clgc.2017.04.012 PMC5771412 28558987

[9] Sedlazeck FJ, Lee H, Darby CA, Schatz MC (2018). Piercing the dark matter: bioinformatics of long-range sequencing and mapping. Nat Rev Genet.

[10] Chaisson MJP, Sanders AD, Zhao X (2019). Multi-platform discovery of haplotype-resolved structural variation in human genomes. Nat Commun.

[11] Zheng GXY, Lau BT, Schnall-Levin M (2016). Haplotyping germline and cancer genomes with high-throughput linked-read sequencing. Nat Biotechnol.

[12] Chen Z, Pham L, Wu T-C, et al (2020) Ultra-low input single tube linked-read library method enables short-read second-generation sequencing systems to generate highly accurate and economical long-range sequencing information routinely. Genome Res gr.260380.119 10.1101/gr.260380.119 PMC7370886 32540955

[13] Marks P, Garcia S, Barrio AM (2019). Resolving the full spectrum of human genome variation using linked-reads. Genome Res.

[14] Viswanathan SR, Ha G, Hoff AM (2018). Structural alterations driving castration-resistant prostate cancer revealed by linked-read genome sequencing. Cell.

[15] Ma X, Liu Y, Liu Y, et al (2018) Pan-cancer genome and transcriptome analyses of 1,699 paediatric leukaemias and solid tumours. Nature. 10.1038/nature25795 10.1038/nature25795 PMC5854542 29489755

[16] Dobin A, Davis CA, Schlesinger F, Drenkow J, Zaleski C, Jha S, Batut P, Chaisson M, Gingeras TR (2013). STAR: ultrafast universal RNA-seq aligner. Bioinformatics (Oxford, England).

[17] 10x Genomics (2018) Longranger software. https://support.10xgenomics.com/genome-exome/software/downloads/latest.

[18] 10x Genomics (2018) Reference genome. http://cf.10xgenomics.com/supp/genome/refdata-GRCh38-2.1.0.tar.gz.

[19] McKenna A, Hanna M, Banks E (2010). The Genome Analysis Toolkit: a MapReduce framework for analyzing next-generation DNA sequencing data. Genome Res.

[20] Ding L-W, Sun Q-Y, Tan K-T (2017). Mutational landscape of pediatric acute lymphoblastic leukemia. Cancer Res.

[21] Sun Q-Y, Ding L-W, Tan K-T (2017). Ordering of mutations in acute myeloid leukemia with partial tandem duplication of MLL (MLL-PTD). Leukemia.

[22] Cibulskis K, Lawrence MS, Carter SL, Sivachenko A, Jaffe D, Sougnez C, Gabriel S, Meyerson M, Lander ES, Getz G (2013). Sensitive detection of somatic point mutations in impure and heterogeneous cancer samples. Nat Biotechnol.

[23] Karczewski KJ, Francioli LC, Tiao G (2020). The mutational constraint spectrum quantified from variation in 141,456 humans. Nature.

[24] Auton A, Abecasis GR, Altshuler DM (2015). A global reference for human genetic variation. Nature.

[25] Ha G, Roth A, Khattra J (2014). TITAN: inference of copy number architectures in clonal cell populations from tumor whole-genome sequence data. Genome Res.

[26] Smigielski EM, Sirotkin K, Ward M, Sherry ST (2000). dbSNP: a database of single nucleotide polymorphisms. Nucleic Acids Res.

[27] Wala JA, Bandopadhayay P, Greenwald NF (2018). SvABA: genome-wide detection of structural variants and indels by local assembly. Genome Res.

[28] Wang K, Li M, Hakonarson H (2010). ANNOVAR: functional annotation of genetic variants from high-throughput sequencing data. Nucleic Acids Res.

[29] Li H, Handsaker B, Wysoker A, Fennell T, Ruan J, Homer N, Marth G, Abecasis G, Durbin R, 1000 Genome Project Data Processing Subgroup (2009). The Sequence Alignment/Map format and SAMtools. Bioinformatics.

[30] Purcell S, Neale B, Todd-Brown K (2007). PLINK: a tool set for whole-genome association and population-based linkage analyses. Am J Hum Genet.

[31] Carrot-Zhang J, Chambwe N, Damrauer JS (2020). Comprehensive analysis of genetic ancestry and its molecular correlates in cancer. Cancer Cell.

[32] DePristo MA, Banks E, Poplin R (2011). A framework for variation discovery and genotyping using next-generation DNA sequencing data. Nat Genet.

[33] Rahman N (2014). Realizing the promise of cancer predisposition genes. Nature.

[34] Zhang J, Walsh MF, Wu G (2015). Germline mutations in predisposition genes in pediatric cancer. N Engl J Med.

[35] Pearl LH, Schierz AC, Ward SE, Al-Lazikani B, Pearl FMG (2015). Therapeutic opportunities within the DNA damage response. Nat Rev Cancer.

[36] Robinson JT, Thorvaldsdóttir H, Winckler W, Guttman M, Lander ES, Getz G, Mesirov JP (2011). Integrative genomics viewer. Nat Biotechnol.

[37] Tate JG, Bamford S, Jubb HC (2019). COSMIC: the Catalogue Of Somatic Mutations In Cancer. Nucleic Acids Res.

[38] Subramanian A, Tamayo P, Mootha VK (2005). Gene set enrichment analysis: a knowledge-based approach for interpreting genome-wide expression profiles. Proc Natl Acad Sci U S A.

[39] Kent WJ, Sugnet CW, Furey TS, Roskin KM, Pringle TH, Zahler AM, Haussler D (2002). The Human Genome Browser at UCSC. Genome Res.

[40] RepeatMasker Web Server. http://www.repeatmasker.org/cgi-bin/WEBRepeatMasker. Accessed 19 May 2021

[41] Ghandi M, Huang FW, Jané-Valbuena J (2019). Next-generation characterization of the Cancer Cell Line Encyclopedia. Nature.

[42] Aguet F, Brown AA, Castel SE (2017). Genetic effects on gene expression across human tissues. Nature.

[43] Love MI, Huber W, Anders S (2014). Moderated estimation of fold change and dispersion for RNA-seq data with DESeq2. Genome Biol.

[44] Li B, Dewey CN (2011). RSEM: accurate transcript quantification from RNA-Seq data with or without a reference genome. BMC Bioinformatics.

[45] Kent WJ (2002). BLAT—The BLAST-like alignment tool. Genome Res.

[46] Greaves DR, Fraser P, Vidal MA, Hedges MJ, Ropers D, Luzzatto L, Grosveld F (1990). A transgenic mouse model of sickle cell disorder. Nature.

[47] Ryan TM, Townes TM, Reilly MP, Asakura T, Palmiter RD, Brinster RL, Behringer RR (1990). Human sickle hemoglobin in transgenic mice. Science.

[48] Ryan TM, Ciavatta DJ, Townes TM (1997). Knockout-transgenic mouse model of sickle cell disease. Science.

[49] Genovese G, Friedman DJ, Ross MD (2010). Association of trypanolytic ApoL1 variants with kidney disease in African Americans. Science.

[50] Giardine B, Borg J, Viennas E (2014). Updates of the HbVar database of human hemoglobin variants and thalassemia mutations. Nucleic Acids Res.

[51] Moore JE, Purcaro MJ, Pratt HE (2020). Expanded encyclopaedias of DNA elements in the human and mouse genomes. Nature.

[52] Nik-Zainal S, Alexandrov LB, Wedge DC (2012). Mutational processes molding the genomes of 21 breast cancers. Cell.

[53] Li Y, Roberts ND, Wala JA (2020). Patterns of somatic structural variation in human cancer genomes. Nature.

[54] Ottaviani D, LeCain M, Sheer D (2014). The role of microhomology in genomic structural variation. Trends Genet.

[55] Jia L, Carlo MI, Khan H (2019). Distinctive mechanisms underlie the loss of SMARCB1 protein expression in renal medullary carcinoma: morphologic and molecular analysis of 20 cases. Mod Pathol.

[56] Torchia J, Golbourn B, Feng S (2016). Integrated (epi)-genomic analyses identify subgroup-specific therapeutic targets in CNS rhabdoid tumors. Cancer Cell.

[57] Biegel JA, Zhou JY, Rorke LB, Stenstrom C, Wainwright LM, Fogelgren B (1999). Germ-line and acquired mutations of INI1 in atypical teratoid and rhabdoid tumors. Cancer Res.

[58] Sullivan LM, Folpe AL, Pawel BR, Judkins AR, Biegel JA (2013). Epithelioid sarcoma is associated with a high percentage of SMARCB1 deletions. Mod Pathol.

[59] Hung RJ, McKay JD, Gaborieau V (2008). A susceptibility locus for lung cancer maps to nicotinic acetylcholine receptor subunit genes on 15q25. Nature.

[60] Kumareswaran R, Ludkovski O, Meng A, Sykes J, Pintilie M, Bristow RG (2012). Chronic hypoxia compromises repair of DNA double-strand breaks to drive genetic instability. J Cell Sci.

[61] Naik RP, Derebail VK, Grams ME (2014). Association of sickle cell trait with chronic kidney disease and albuminuria in African Americans. Jama.

[62] Moltke I, Albrechtsen A, Hansen TVO, Nielsen FC, Nielsen R (2011). A method for detecting IBD regions simultaneously in multiple individuals--with applications to disease genetics. Genome Res.

[63] Pinto EM, Billerbeck AEC, Villares MCBF, Domenice S, Mendonça BB, Latronico AC (2004). Founder effect for the highly prevalent R337H mutation of tumor suppressor p53 in Brazilian patients with adrenocortical tumors. Arq Bras Endocrinol Metabol.

[64] Wen-Chi H, Nair AK, Sayuko K, Peng C, Göring Harald HH, Pollin TI, Alka M, Knowler WC, Baier LJ, Hanson RL (2017). Identity-by-descent mapping identifies major locus for serum triglycerides in Amerindians largely explained by an APOC3 founder mutation. Circ Cardiovasc Genet.

[65] Letouzé E, Sow A, Petel F, Rosati R, Figueiredo BC, Burnichon N, Gimenez-Roqueplo A-P, Lalli E, de Reyniès A (2012). Identity by descent mapping of founder mutations in cancer using high-resolution tumor SNP data. PLoS One.

[66] Lin R, Charlesworth J, Stankovich J, Perreau VM, Brown MA, Taylor BV, Consortium Anz (2013). Identity-by-descent mapping to detect rare variants conferring susceptibility to multiple sclerosis. PLoS One.

[67] Harold D, Connolly S, Riley BP (2019). Population-based identity-by-descent mapping combined with exome sequencing to detect rare risk variants for schizophrenia. Am J Med Genet B Neuropsychiatr Genet.

[68] Albrechtsen A, Sand Korneliussen T, Moltke I, van Overseem Hansen T, Nielsen FC, Nielsen R (2009). Relatedness mapping and tracts of relatedness for genome-wide data in the presence of linkage disequilibrium. Genet Epidemiol.

[69] Raelson JV, Little RD, Ruether A (2007). Genome-wide association study for Crohn’s disease in the Quebec Founder Population identifies multiple validated disease loci. Proc Natl Acad Sci.

[70] Loh P-R, Danecek P, Palamara PF (2016). Reference-based phasing using the Haplotype Reference Consortium panel. Nat Genet.

[71] Browning SR, Browning BL (2007). Rapid and accurate haplotype phasing and missing-data inference for whole-genome association studies by use of localized haplotype clustering. Am J Hum Genet.

[72] Delaneau O, Marchini J, Zagury J-F (2012). A linear complexity phasing method for thousands of genomes. Nat Methods.

[73] Choi Y, Chan AP, Kirkness E, Telenti A, Schork NJ. Comparison of phasing strategies for whole human genomes. PLoS Genet. 2018; 10.1371/journal.pgen.1007308. 10.1371/journal.pgen.1007308 PMC5903673 29621242

[74] Sharp K, Kretzschmar W, Delaneau O, Marchini J (2016). Phasing for medical sequencing using rare variants and large haplotype reference panels. Bioinformatics.

[75] Browning SR, Thompson EA (2012). Detecting rare variant associations by identity-by-descent mapping in case-control studies. Genetics.

[76] Hong AL (2021) Genomics of pediatric renal medullary carcinomas. https://www.ncbi.nlm.nih.gov/projects/gap/cgi-bin/study.cgi?study_id=phs001800.v2.p1.

