# Supplementary material for: Haplotype-resolved germline and somatic alterations in renal medullary carcinomas
Source: Genome Med. 2021 Jul 14;13:114. doi: 10.1186/s13073-021-00929-4 (PMC8281718; doi:10.1186/s13073-021-00929-4)
Supplement: Supplementary file 2 — Additional file 2: Figures S1-S5. Supplementary figures and their corresponding figure legends. [file 13073_2021_929_MOESM2_ESM.docx]

# **Supplementary Figures**


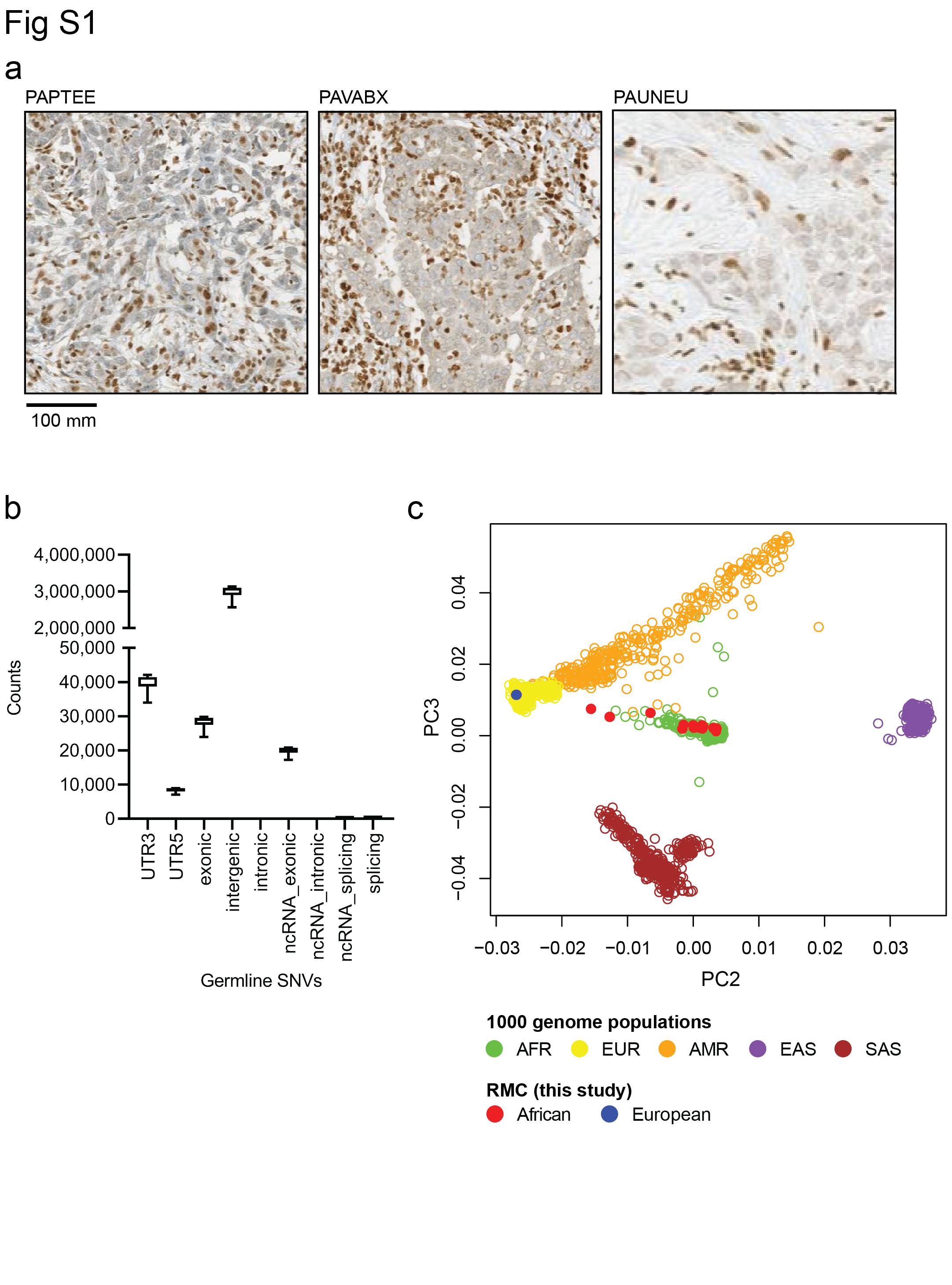


**Figure S1: Summary of germline variants and inferred ancestry of RMC patients. (a)** Representative SMARCB1 immunohistochemistry (brown) from patients with RMC. Rhabdoid cells demarking loss of SMARCB1 expression. **(b)** Counts of germline single nucleotide variants in different regions of the genome in 11 matched and 1 unmatched normal samples. Germline variants were classified based on their location relative to annotated genes and transcripts. **(c)** Principal component analysis of germline genotype calls for RMC and the 1000 Genome Project phase 3 samples. The second and third principal components (PC2 and PC3) effectively separates the 1000 Genome Project samples into the 5 major populations representing Africans (AFR), Ad Mixed Americans (AMR), East Asian (EAS), Europeans (EUR), and South Asian (SAS). Both normal and tumor RMC patient samples were included in the plot. RMC patients self-reported as African and European ancestry were separately labelled.

**
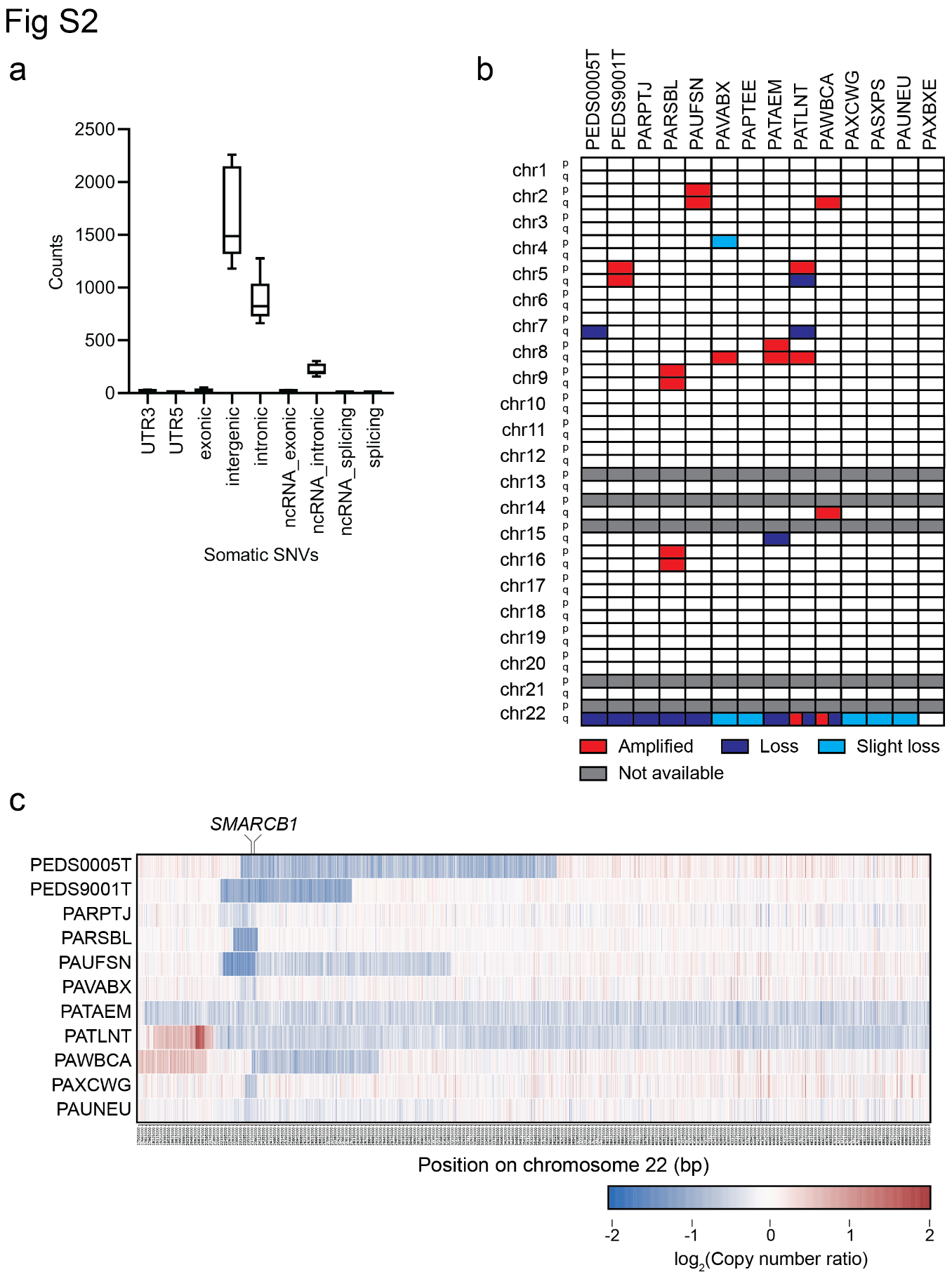
**

**Figure S2: Summary of somatic single-nucleotide variants (SNVs) and copy number alterations detected in renal medullary carcinoma samples. (a)** Counts of somatic single nucleotide variants in different regions of the genome in 11 tumor samples with matched normal controls are as indicated. **(b)** Heatmap depicting copy number alterations identified in each chromosome arm in 11 tumor samples with matched germline samples and 3 tumor samples with unmatched germline samples. Arms are noted as regions with ‘loss’ if copy number loss were identified by the TITAN algorithm. Arms were indicated as regions with ‘slight loss’ if loss events were not automatically identified as regions with copy number losses by the TITAN algorithm, but where copy number losses could still be visibly observed from the copy number and allelic ratio plots. Specifically, these generally represent regions where copy number losses were slight (log ratio ~ -0.5), and/or regions where the deletions were focal. The acrocentric chromosomal arms which are represented as gaps in the reference genome were not analyzed and indicated as ‘not available’. **(c)** Copy number gain and losses observed on chromosome 22. Region where *SMARCB1* resides is highlighted.

**
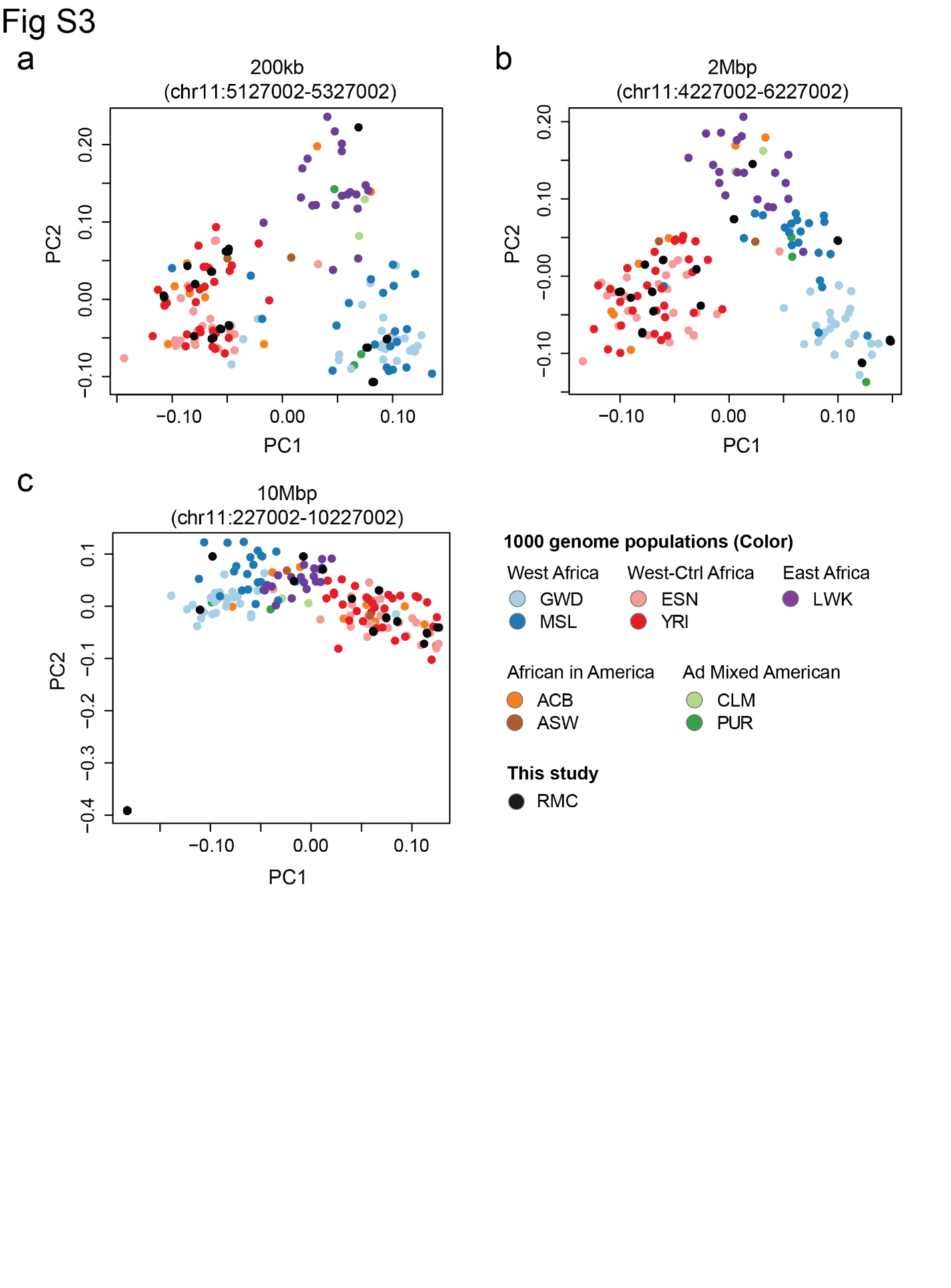
**

**Figure S3: Non-haplotype level principal component analysis of the *HBB* locus.** Principal component analysis was performed for the 14 RMC patients of African ancestry, together with individuals with sickle cell trait from the 1000 Genomes Project (phase 3). This analysis was performed for regions of different sizes representing **(a)** 200kb, **(b)** 2Mbp, and **(c)** 10 Mbp around the sickle cell mutation.

**
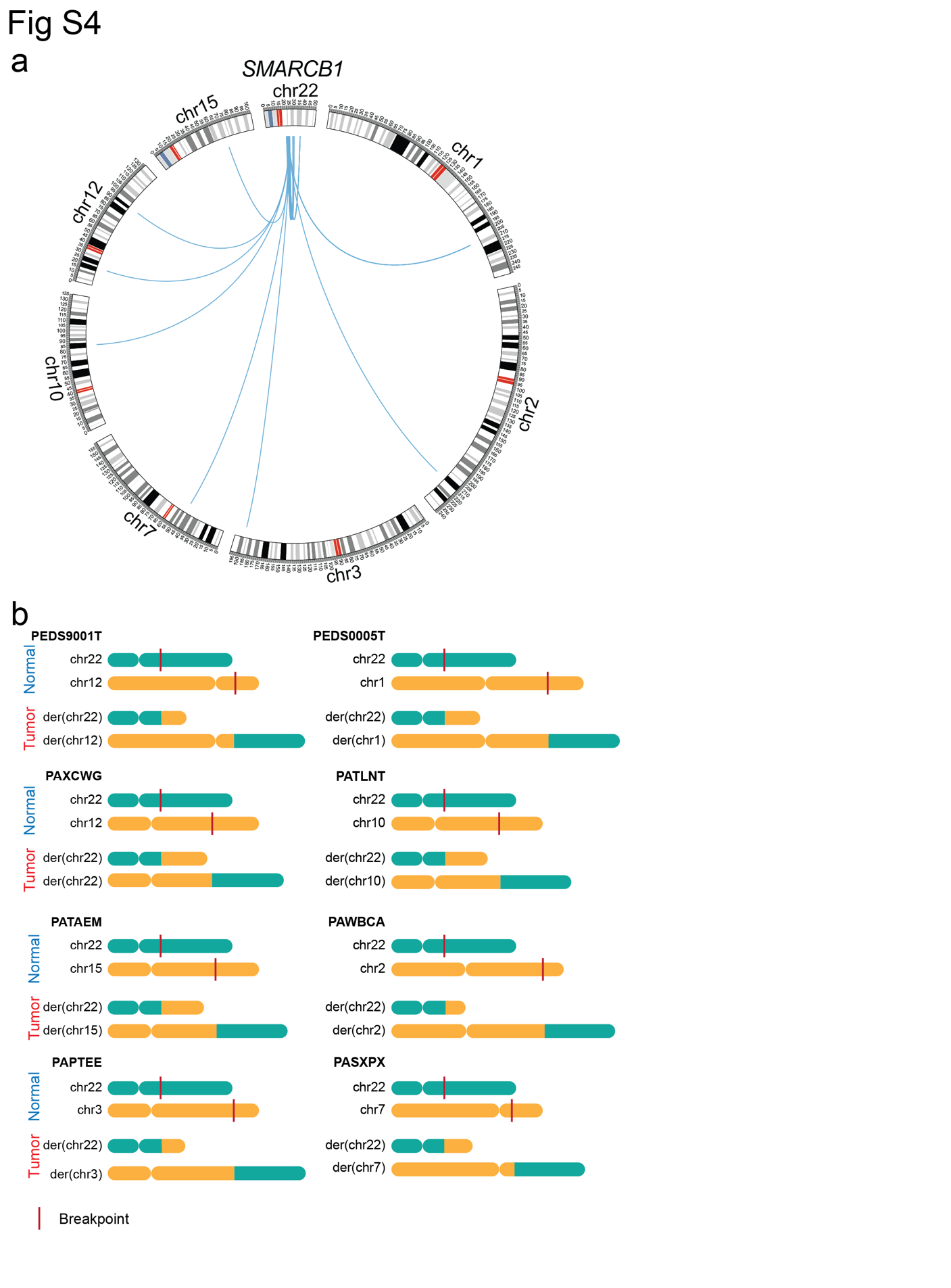
**

**Figure S4: Structures of *SMARCB1* translocations and deletions in RMC samples.** **(a)** Circos plot depicting locations of *SMARCB1* translocation and deletion events. Each of the blue lines highlights the pair of genomic coordinates representing a *SMARCB1* chromosomal translocation or large deletion event. **(b)** Chromosomal structures of *SMARCB1* related translocations. Structures of derived chromosomes were inferred based on structural variants detected in the *SMARCB1* gene.

**
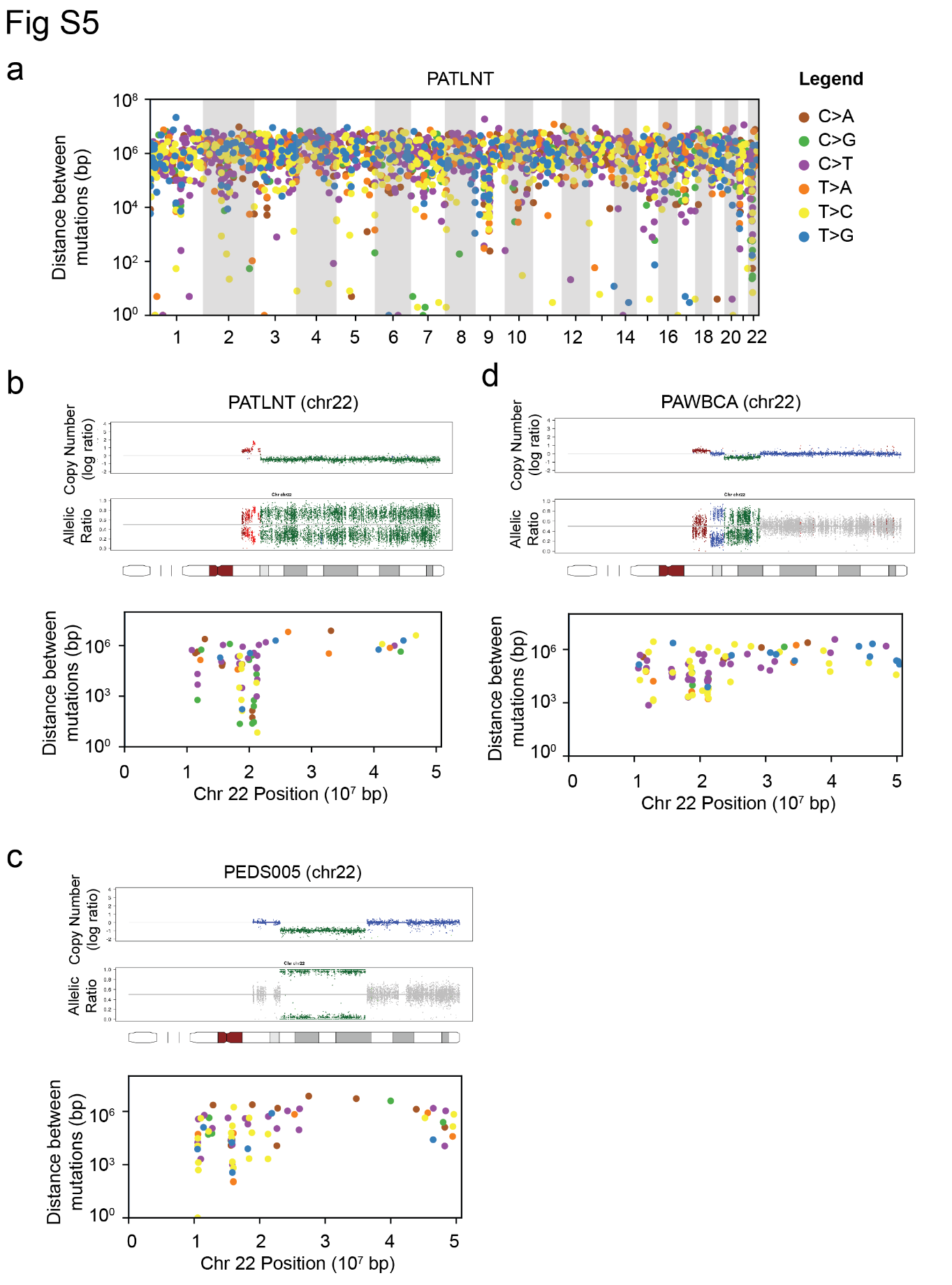
**

**Figure S5: Rainfall plots for somatic single nucleotide mutations in RMC samples.** **(a)** Genome-wide rainfall plot for PATLNT identifies areas of potential kataegis in chromosomes 9 and 22. **(b)** Detailed analysis of chromosome 22 in patient sample PATLNT along with copy number alterations as identified by TITAN show an association of increased mutations with changes in copy number. (**c-d**) Additional examples of kataegis identified in RMC samples.
